# Special Issue ‘Advanced and High Performance Metallic Foams’

**DOI:** 10.3390/ma14051092

**Published:** 2021-02-26

**Authors:** Imre Norbert Orbulov

**Affiliations:** 1 Department of Materials Science and Engineering, Faculty of Mechanical Engineering, Budapest University of Technology and Economics, Műegyetem rakpart 3., 1111 Budapest, Hungary; orbulov@eik.bme.hu; 2 MTA–BME Lendület Composite Metal Foams Research Group, Műegyetem rakpart 3., 1111 Budapest, Hungary

Metallic foams are developing more and more. Nowadays, advanced or high-performance metallic foams (such as low-cost, high-strength, high-stiffness, excellent energy absorbers) are emerging. Researchers in the metallic-foam community are pushing the boundaries further and further to produce stronger, lighter, higher-performance, or functional foams for special applications.

The Special Issue entitled “Advanced and High Performance Metallic Foams” covers all types and aspects of metallic foams from design through production and intensive testing, The issue includes, but is not limited to, liquid and solid-state and 3D additive production methods; blowing agents; foaming; macro-, meso-, and micro-structures of foams; structural reverse engineering; modelling of metallic foams; quasi-static, dynamic and cyclic mechanical properties (compressive, tensile, and bending behavior, including blast protection and piercing) at room, elevated, or cryogenic temperature; mechanical damping; failure mechanisms and energy absorption of metallic foams; foam-filled structures and their mechanical stability; biocompatible foams; degradable and recyclable metallic foams; joining technologies like brazing, welding, and gluing; forming of metallic foams; notch and hole sensitivity; applications; and case studies.

The first main line of the manuscript focuses on the basic (compressive) mechanical properties of the foams. Advanced metal matrix syntactic foams (MMSFs) were widely studied in this aspect. Mohavedi et al. [1] produced and investigated zinc alloy (ZA27) syntactic foams (SFs), manufactured using expanded perlite (EP) particles and liquid state counter-gravity infiltration casting. As-cast and solution heat-treated samples were tested to investigate the compressive properties of the ZA27 syntactic foam. Quasi-static compression tests and microstructural analysis of the as-cast and heat-treated syntactic foams were carried out using scanning electron microscopy (SEM). The heat treatment altered the microstructure of the ZA27 alloy matrix from a multiphase dendrite to a spheroidized microstructure with improved ductility. Moreover, the heat treatment considerably enhanced the energy absorption and plateau stress of the MMSFs. Kádár et al. [2] extensively applied acoustic emission methods to describe the damage and deformation mechanisms of a liquid -state, inert-gas pressure infiltration-produced aluminum matrix, expanded perlite filled MMSFs. The dominant deformation mechanisms during compression of the foam were determined by sequential k-means analysis of the acoustic emission data. Since the different deformation mechanisms were concurrently active even at small strains, successive unloading and reloading measurement was proposed for cluster identification. The repetitive unloading and reloading permitted the identification of two significant influencing mechanical parameters, namely, the unloading modulus and the loss for unloading-reloading cycles. Sticking to the MMSFs, Szlancsik et al. [3] studied the effect of different filler materials from a mechanical point of view and an economic aspect as well (since one of the main obstacles to the widespread use of MMSFs is cost). Three different filler materials: (i) ceramic hollow spheres (CHSs), (ii) metallic hollow spheres (MHSs) and (iii) lightweight expanded clay particles (LECAPs), have been investigated in various aspects (microstructure, mechanical properties, cost). In the terms of cost-awareness the LECAPs are the best fillers, because they are ~100 times cheaper than the CHSs or MHSs, but their mechanical properties can be compared to the aforementioned, relatively expensive filler materials and still exceed the properties of the most “conventional” metallic foams (produced by foaming agents or by templating, for example). Regarding the “conventional” aluminum foams, Amaro et al. [4] studied aluminum alloy foams produced by the lost wax casting process, with nominal relative densities of 20%, 40%, and 60% as well as arrangements of a uniform cell structure (US) or a dual-size cell (DS). Impact tests at different velocities were performed on the samples using a split Hopkinson pressure bar (SHPB). All of the abovementioned publications provide important data on the properties of MMSFs and conventional foams.

Besides the conventional compressive tests, advanced materials testing methods such as fracture mechanics, are considered. Lehmhus et al. [5] and Szlancsik et al. [6] studied the fracture toughness of MMSFs containing hollow spheres as filler in different matrices (steel and aluminum) with respect to notch sensitivity. This was studied via (i) elastic-plastic fracture mechanics measurements (determination of R-curves based on three-point bending tests) and (ii) Charpy impact tests. The findings can be connected to the characteristics of the deformation zone and the associated stress concentration at the tip of the machined notches.

Advanced metallic foams can be produced by the direct engineering of their pore structure as well. Borovinšek et al. [7] reports on a detailed investigation of external (e.g., shape and size) and internal (e.g., distribution, size, number of pores) geometry and porosity changes of Advanced Pore Morphology (APM) foam elements, during compressive loading by means of the ex-situ micro-Computed Tomography (CT), and advanced digital image analysis and recognition. The results show that the porosity of APM foam elements decreased by only 25% at the engineering strain of 70% due to an increase of the number of pores at high stages of compressive deformation. The APM foam elements also exhibited a positive macroscopic Poisson’s ratio of 0.2, which is uncharacteristic of cellular structures. Vesenjak et al. [8] published a study on the development of a new unidirectional cellular (UniPore) copper structure with multiple concentric pipe layers. The investigated UniPore structures were grouped into three main types, each having a different number of pipes (3, 4, and 5 per transversal cross-section) and different pore arrangements. The specimens were fabricated by explosive compaction to achieve tightly compacted structures with a quasi-constant cross-section along the length of the specimens. The bonding between copper pipes was observed by a metallographic investigation, which showed that the pipes and bars were compressed tightly without voids but not welded together. The mechanical properties were determined by quasi-static compressive testing, where the typical behavior for cellular materials was noted. The study showed that porosity significantly influences the mechanical properties even more so than the arrangement of the pipes.

The Special Issue also published three interesting papers on metallic foams for special applications and functions. Liang et al. [9] studied the blast resistance of a sandwich-walled cylinder/ring comprising two metal face-sheets and a graded metal foam core, subjected to internal air blast loading. The deformation process was divided into three distinct phases: fluid–structure interaction, core-crushing, and outer face-sheet deformation. Finite element modeling was performed using the Voronoi material model. The proposed analytical models were verified through finite element analysis and reasonable agreement was observed between the analytical predictions and finite element results. The results indicated that both the deformation modes and the structural response of the cylinders are sensitive to the blast charge and core configuration. This in-depth understanding of the behavior in sandwich-walled cylinders under blast impulse and the influence of the core configuration helped illustrate the advantages and disadvantages of using graded foam materials in sandwich structures and provided a guideline for structural design. Wang et al. [10] prepared CuO directly on copper foam substrate by anodic oxidation. The effects of current density and anodizing temperature on sample preparation and performance were studied. Field emission scanning electron microscopy (FESEM) and an X-ray diffractometer (XRD) had been used to determine the morphology and phase structure of the sample, and its optical and electrical properties were discussed through UV-vis spectrophotometer and electrochemical tests with the aim of solar energy conversion applications. Dyga and Płaczek [11] investigated the air-water and air-oil two-phase flow patterns in channels with open-cell metal foams. The analysis applied three foams with pore density equal to 20, 30 and 40 pore per inch (PPI). Plug flow, slug flow, stratified flow and annular flow were observed over the ranges of gas and liquid surfaces. Churn flow, which had not yet been observed in the flow through the open–cell foams, was also recorded. A new gas−liquid flow pattern map for a channel packed with open-cell metal foams with high porosity was developed. The map is valid for liquids with a density equal to or lower than the density of water and a viscosity several times greater than that of water.

Last but not least, a study on the machinability of foams was published by Silva et al. [12]. The machining of cellular metals has been a challenge, as the resulting surface is extremely irregular, with torn off or smeared material and poor accuracy. In their study, a 2D finite element model of peripheral milling for cellular metals was presented. The model was able to simulate chip separation as well as surface and subsurface damage on the machined surface. Although the calculated average cutting force was not accurate, the model provided a reasonable estimation of maximum cutting force.

In summary, the main focus of foam-related publications is still in their mechanical properties. Newer and newer foam structures are suggested, and newer and newer aspects are revealed to enhance the usability and spreading of metallic foams. More configurations mean more possibilities and wider application windows. On the other hand, the special applications of foams are approached through their functional properties (blast protection, flow patterns, high surface substrates in the examples of this Special Issue). These case studies reveal important application possibilities as well.

## References

[1] Movahedi N., Murch G.E., Belova I.V., Fiedler T. (2019). Effect of Heat Treatment on the Compressive Behavior of Zinc Alloy ZA27 Syntactic Foam. Materials.

[2] Kádár C., Chmelík F., Ugi D., Máthis K., Knapek M. (2019). Damage Characterization during Compression in a Perlite-Aluminum Syntactic Foam. Materials.

[3] Szlancsik A., Katona B., Kemény A., Károly D. (2019). On the Filler Materials of Metal Matrix Syntactic Foams. Materials.

[4] Amaro A.M., Neto M.A., Cirne J.S., Reis P.N.B. (2019). Mechanical Characterization of Different Aluminium Foams at High Strain Rates. Materials.

[5] Lehmhus D., Weise J., Szlancsik A., Orbulov I.N. (2020). Fracture Toughness of Hollow Glass Microsphere-Filled Iron Matrix Syntactic Foams. Materials.

[6] Szlancsik A., Katona B., Károly D., Orbulov I.N. (2019). Notch (In) Sensitivity of Aluminum Matrix Syntactic Foams. Materials.

[7] Borovinšek M., Vesenjak M., Higa Y., Shimojima K., Ren Z. (2019). Characterization of Geometrical Changes of Spherical Advanced Pore Morphology (APM) Foam Elements during Compressive Deformation. Materials.

[8] Vesenjak M., Nakashima Y., Hokamoto K., Ren Z., Marumo Y. (2020). Development of Unidirectional Cellular Structure with Multiple Pipe Layers and Characterisation of Its Mechanical Properties. Materials.

[9] Liang M., Li X., Lin Y., Zhang K., Lu F. (2020). Theoretical Analysis of Blast Protection of Graded Metal Foam-Cored Sandwich Cylinders/Rings. Materials.

[10] Wang B., Cao B., Wang C., Zhang Y., Yao H., Wang Y. (2020). The Optical and Electrical Performance of CuO Synthesized by Anodic Oxidation Based on Copper Foam. Materials.

[11] Dyga R., Płaczek M. (2020). Influence of Hydrodynamic Conditions on the Type and Area of Occurrence of Gas–Liquid Flow Patterns in the Flow through Open–Cell Foams. Materials.

[12] Silva R.G., Teicher U., Brosius A., Ihlenfeldt S. (2020). 2D Finite Element Modeling of the Cutting Force in Peripheral Milling of Cellular Metals. Materials.

